# Intercepting the Intussusception: A Rare Case of Adult Intussusception With a Cecal Neuroendocrine Tumor Lead Point

**DOI:** 10.7759/cureus.64460

**Published:** 2024-07-13

**Authors:** Ermal Hasalliu, Kaiser F Kabir, Jeremy Hess, Julia Greer

**Affiliations:** 1 Internal Medicine, Ascension Macomb-Oakland Hospital, Detroit, USA; 2 Internal Medicine, Michigan State University College of Osteopathic Medicine, Warren, USA; 3 Internal Medicine, Ascension Macomb-Oakland Hospital, Warren, USA; 4 Gastroenterology, Ascension Providence Hospital, Southfield, USA

**Keywords:** small bowel resection, bowel obstruction, idiopathic adult intussusception, intussusception lead point, ileocecal intussusception, neuroendocrine neoplasm, intussusception in the elderly

## Abstract

Intussusception is the invagination of one segment of the bowel into the adjacent bowel segment leading to obstruction, intestinal ischemia and, in severe cases, peritonitis and perforation. While the condition is more common in children, adult intussusception does occur and is often attributed to malignancy. In this case report, we discuss an adult man who presented for weight loss and intermittent abdominal pain and was ultimately found to have ileocecal intussusception on CT imaging. A colonoscopy with cold biopsy was performed and pathology reports displayed a well-differentiated neuroendocrine tumor lead point; a rare event with only a few cases reported. Ultimately, the patient was taken to the operating room, and an ileocecectomy was performed with primary anastomosis. Prompt diagnosis and management are crucial in adult intussusception as a missed event can lead to tumor progression, bowel ischemia, bleeding and necrosis.

## Introduction

Intussusception is the invagination of one segment of the bowel into the intraluminal layer of the adjacent bowel segment leading to an obstruction, intestinal ischemia and, in severe cases, peritonitis and perforation. Most cases of intussusception are found in children and are idiopathic in nature without an identifiable lead point. However, adult intussusception (AI) represents 5% of all cases and accounts for only 1-5% of intestinal obstructions in adults [1]. While up to 20% of adult intussusception cases are idiopathic and localized to the small intestine; secondary etiologies are indolent and caused by pathologic mechanisms such as carcinoma, diverticular disease, polyps or benign neoplasms. In this case report, we discuss an adult man with ileocecal intussusception who was found to have a well-differentiated neuroendocrine tumor lead point.

## Case presentation

**Figure 1:**
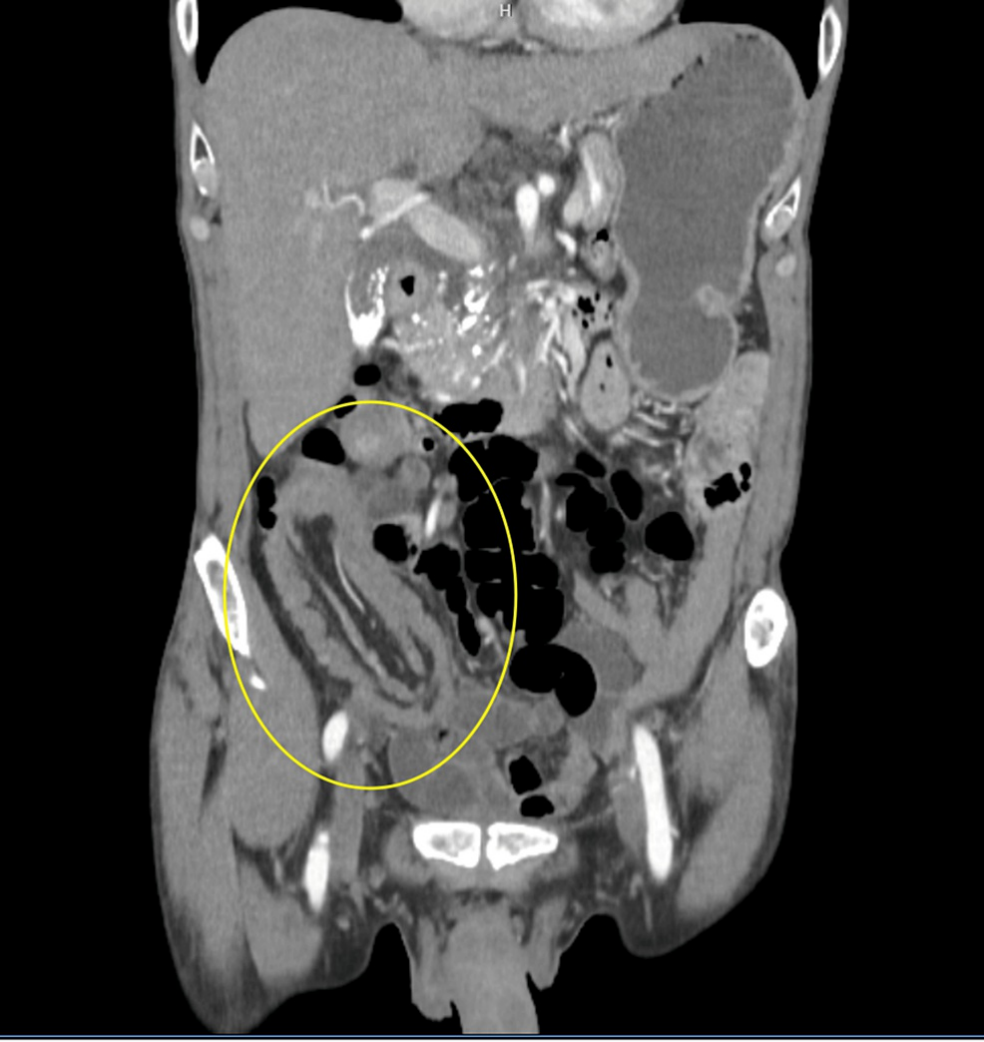
Ileocecal intussusception with cecal soft tissue thickening found on CT imaging (Yellow circle)

​​A 65-year-old African-American man with chronic pancreatitis, alcohol use disorder and hypertension came into the hospital after outpatient evaluation for weight loss. He was instructed by his primary care physician to be evaluated at the hospital because of unintentional weight loss. An outpatient CT Abdomen was performed and demonstrated intussusception along with a rectal mass. Upon emergency department arrival, the patient had no acute complaints but recalled periumbilical abdominal pain the last 5-7 days. The pain occurred occasionally without any noticeable triggers and he defined it as a "cramping" sensation with 4/10 intensity. In addition, he experienced abdominal distention with one episode of non-bilious, non-bloody vomiting. After symptom resolution, the patient denied any further abdominal pain, and on admission denied constipation or alcohol use in the aforementioned time frame. The patient did confirm unintentional weight loss in the last few months, of approximately 35-40 lbs. In the ER, another CT abdomen/pelvis with contrast was performed which demonstrated a long segment of ileocecal intussusception and associated cecal soft tissue thickening suggestive of a neoplastic lead point (Figure 1).

**Figure 2:**
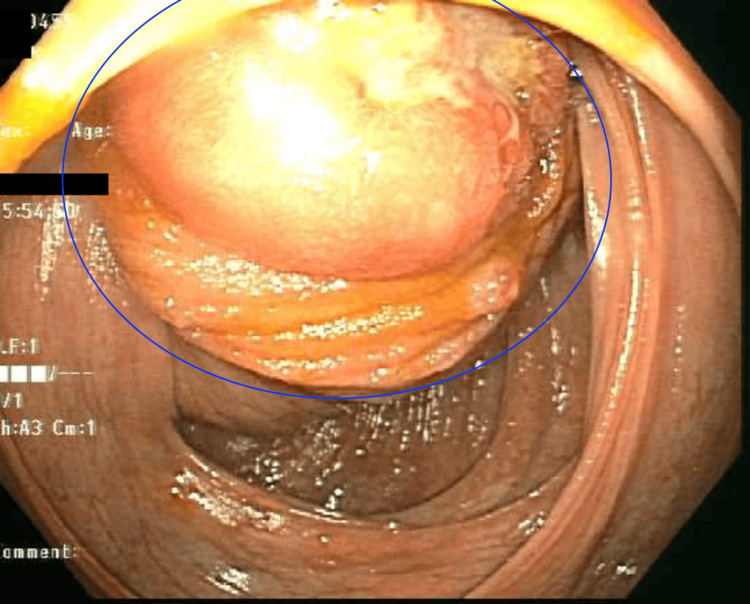
Endoscopic view of the cecal mass (blue circle)

A colonoscopy was performed which found a large, ulcerated and completely obstructive mass in the cecum, with an approximate diameter of 30 mm (Figure 2). At this point, the colonoscope could not be advanced and biopsies of the mass were obtained. The pathology report showed a well-differentiated neuroendocrine neoplasm partly involving inflamed and distorted enteric mucosa. Ultimately, the patient was taken to the operating room, and an ileocecectomy was performed with primary anastomosis. Tumor markers CEA was 4.0 (ref. range 0-3.4 ng/mL) and CA19-9 was 68 (ref range 0-37 unit/mL). Pathology from the surgical biopsy revealed a well-differentiated neuroendocrine tumor with invasion into the muscularis propria into the subserosa. There was metastatic disease in three of 14 lymph nodes, ultimately described as T3 N1 disease. After the first postoperative day, the patient was able to advance diet and was having normal bowel movements. He was discharged in stable condition and largely asymptomatic. He was instructed to establish care with oncology on an outpatient basis.

## Discussion

Gastrointestinal neuroendocrine tumors vary from slow-growing tumors with distinct histological characteristics to poorly differentiated carcinomas. While there are four types of neuroendocrine tumors, small intestinal neuroendocrine tumors are the most common small bowel malignancy and most are located in the terminal ileum within 60 cm of ileocecal valve. As the size of the tumor increases above 1.1 cm the tendency to metastasize increases from 2% to 28% [2]. In addition, with increasing cell burden, their physical manifestation takes form along with their complications. Adult intussusception (AI) is a notable complication, which is induced via irritation of intestinal peristalsis utilizing tumor burden as a lead point.

AI occurs most often in the small bowel and is categorized according to location with 39% enteroenteric, 13% ileocolic and 17% ileocecal (ileocecal valve is the lead point) [3]. Multiple case series indicate that over 50% of colonic lesions are malignant in nature, primarily adenocarcinoma, whereas metastasis was the most common malignant lead point in the small intestine [4]. Other well-established pathologic mechanisms include polyps, diverticula, Meckel diverticulum, stenosis, or benign neoplasms. In rare cases, as in our patient, the intussusception lead point is a nonfunctional neuroendocrine tumor. Neuroendocrine tumor lead points are rare with only a handful of cases reported with most cases appearing as functional carcinoid neuroendocrine tumor [5-8]. 

Presentation of AI varies with patients presenting with symptoms of small or large bowel obstruction including intermittent abdominal pain, bloating, nausea and vomiting, similar to our patient. As the invagination between the intraluminal layers advances, the probability of impinging blood supply leading to bowel ischemia increases; ultimately increasing the risk of gastrointestinal bleeding and necrosis [9, 10, 11]. 

With varying clinical presentation, the most sensitive diagnostic method of AI is abdominal CT, especially in patients with non-specific abdominal pain. Common CT abdomen findings include the target sign, the reniform pattern, and the sausage-shaped pattern which are used to represent the different phases of intussusception from early stage to late stages respectively. Another modality for diagnosis is abdominal multidetector computed tomography (MDCT) which has been found to display an accuracy of 58-100% in detecting adult intussusception [12]. It is utilized not only to delineate the presence of the disease but also the location, intestinal segments involved and the presence or lack of a leading point. In addition, MDCT can demonstrate the complications of intussusceptions, most notably bowel wall ischemia and perforation. While in children the modality of choice is abdominal ultrasound which shows the target and doughnut sign.

Colonoscopy can also be used as a tool for evaluating AI and underlying masses, especially in those with concern for a large bowel obstruction. Retrospective studies demonstrate that colonoscopy was 100% accurate in diagnosing intussusception compared to CT (90% accuracy) [13]. In addition, colonoscopy not only allows for direct visualization of an underlying mass but also for biopsy. Studies demonstrate that patients with colon cancer found via colonoscopy had a lower-stage of disease on presentation and better outcomes albeit due to the timing of tumor identification along with assessing tumor size [14]. While colonoscopy has a high diagnostic accuracy of AI and underlying masses, in rare cases colonoscopy-induced intussusception has also occurred. The exact mechanism is unknown, but Lasithiotakis et al. hypothesize that aspiration of insufflated air along with hyperperistaltic movement of the small intestine can lead to the formation of a lead point and intussusception [15]. 

In the pediatric population, the treatment modality for intussusception is ultrasound-guided or fluoroscopic pneumatic or hydrostatic enema, and is successful in 85-90% of cases. While in adults, due to the indolent nature of intussusception, exploratory laparotomy followed by resection of lead point masses or areas of ischemia is necessary. As performed with our patient, post-op pathological sample analysis is essential in diagnosing any underlying malignancy, especially in older patients. 

## Conclusions

Adult intussusception is a rare condition with variable clinical presentation with patients complaining of abdominal pain and bloating. Due to its broad clinical presentation, abdominal CT is the most sensitive imaging modality in making a diagnosis and defining the presence of lead points. Ultimately the management in adults is surgical laparotomy to isolate the lead point which can later on be analyzed for malignancy. Prompt diagnosis and management are crucial in adult intussusception as a missed event can lead to tumor progression, bowel ischemia, bleeding, and necrosis.
